# EEG-Based Intersubject Correlations Reflect Selective Attention in a Competing Speaker Scenario

**DOI:** 10.3389/fnins.2021.685774

**Published:** 2021-06-14

**Authors:** Marc Rosenkranz, Björn Holtze, Manuela Jaeger, Stefan Debener

**Affiliations:** ^1^Neuropsychology Lab, Department of Psychology, University of Oldenburg, Oldenburg, Germany; ^2^Division Hearing, Fraunhofer Institute for Digital Media Technology IDMT, Speech and Audio Technology, Oldenburg, Germany; ^3^Cluster of Excellence Hearing4all, University of Oldenburg, Oldenburg, Germany; ^4^Research Center for Neurosensory Science, University of Oldenburg, Oldenburg, Germany

**Keywords:** intersubject correlation, EEG, attended speaker paradigm, naturalistic stimuli, speech envelope tracking, selective auditory attention, correlated component analysis

## Abstract

Several solutions have been proposed to study the relationship between ongoing brain activity and natural sensory stimuli, such as running speech. Computing the intersubject correlation (ISC) has been proposed as one possible approach. Previous evidence suggests that ISCs between the participants’ electroencephalogram (EEG) may be modulated by attention. The current study addressed this question in a competing-speaker paradigm, where participants (*N* = 41) had to attend to one of two concurrently presented speech streams. ISCs between participants’ EEG were higher for participants attending to the same story compared to participants attending to different stories. Furthermore, we found that ISCs between individual and group data predicted whether an individual attended to the left or right speech stream. Interestingly, the magnitude of the shared neural response with others attending to the same story was related to the individual neural representation of the attended and ignored speech envelope. Overall, our findings indicate that ISC differences reflect the magnitude of selective attentional engagement to speech.

## Introduction

The effects of attention on the neural processing of the human brain are typically studied with discrete and highly controlled stimuli. While this has certainly contributed to the understanding of information processing in the brain, recently, paradigms have shifted toward more ecologically valid designs that mirror real-world scenarios. One naturalistic study design is the attended speaker paradigm, in which participants are instructed to attend to one of two (or more) simultaneously presented speech streams (Cherry, 1953). Over the past few years several methods have been developed to study how the brain deals with the complexity and dynamics of running speech (Hamilton and Huth, 2020). One such method is the intersubject correlation (ISC) which calculates the correlation across participants’ brain signals to assess the reliability of the brain response between participants. ISCs were first established in functional neuroimaging and revealed important insights into commonalities in sensory processing (Hasson et al., 2004). While discrete stimuli require repeated presentation to acquire a reliable response (Luck, 2014), ISC has the advantage that a single exposure of the same continuous stimulus to each participant is sufficient to produce a measurable brain response across participants.

Shared brain responses as captured by ISC contain lower-level sensory processes as well as higher-level processes such as memory retention (Hasson et al., 2004, 2008; Cohen and Parra, 2016). Previously, ISC of electroencephalographic (EEG) signals have been indicated to be influenced by attention (Dmochowski et al., 2012; Ki et al., 2016; Cohen et al., 2018). When diverting attention away from a sensory stream, the resulting ISC will be smaller across participants (Ki et al., 2016; Cohen et al., 2018). Accordingly, the allocation of attention to the stimulus increases ISCs. So far, single stream paradigms have been used to study attention effects on EEG-derived ISCs. In such a case, as a control condition, attention is diverted away from the primary task by secondary task demands. However, this approach may come with several limitations. Firstly, single streams such as movies or audiobooks (presented in the absence of background noise) require only modest attentional resources (Herrmann and Johnsrude, 2020). Secondly, dual task manipulations may not only divert attention away from the primary task but may also impact on sensory processing and memory demands (Lavie, 2005). This limits the exclusive attribution of single vs. dual task differences to attention. Besides, a validation of ISC values as being influenced by attention could be supported by correlating these effects to well established neural signatures of selective attention, such as speech envelope tracking (Kerlin et al., 2010; Ding and Simon, 2012; Horton et al., 2013; Zion Golumbic E. M. et al., 2013; Kong et al., 2014). The aim of the present study was to address these issues by using a competing-speaker paradigm. In this challenging listening scenario attentional resources are required to comprehend the target speaker, and sensory stimulation and memory demands are identical, regardless of whether individuals are instructed to attend to the left or right speech stream.

In the current study, participants were presented with two stories simultaneously and attended to one of them while their EEG was recorded. Importantly, all participants were exposed to the same stimulus, i.e., both stories. Stimuli were not repeated, because repetition can reduce ISC values (Dmochowski et al., 2012; Ki et al., 2016; Cohen et al., 2017). We hypothesized that ISC values between participants attending to the same story are higher than ISC values between participants attending to different stories. We reasoned that ISC values across opposing conditions reflect the shared neural response to the physical properties of the stimulus. Thus, the difference between ISC values of participants within the same condition and across conditions would reflect the attention effect.

To further explore the individual differences of attentional focus, ISC was related to speech envelope tracking, which is a well-established procedure measuring the participants’ neural response to running speech (Ding and Simon, 2012). One property of running speech is its temporal fluctuations in amplitude, known as the speech envelope (Rosen, 1992), which is reflected in the human auditory cortex when listening to running speech (Aiken and Picton, 2008; Kubanek et al., 2013). When multiple speech streams are presented concurrently, selective attention to one of them seems to act as a top-down sensory gain control mechanism. This mechanism enhances the responses to the attended auditory stimulus relative to the ignored stimulus. It is reflected in a stronger correlation between the EEG activity and the attended speech envelope compared to the ignored speech envelope (Ding and Simon, 2012; Horton et al., 2013; Kong et al., 2014). The strength of the attentional gain on speech envelope tracking hints at whether a participant comprehended the to-be-attended speaker (O’Sullivan et al., 2015). Given that ISC as well as speech envelope tracking are modulated by attention it was expected that ISCs of participants attending to the same story are positively related to the attention effects on speech envelope tracking.

Lastly, ISC was used to predict whether participants attended to the left or right story. Therefore, the analysis of Ki et al. (2016) was adapted. In contrast to Ki et al. (2016), no second task was necessary as attending to one story automatically resulted in diverting attention from the other story. Furthermore, attending to multiple streams compared to one stream is more demanding. Thus, by comparing prediction accuracies between the two studies, differences in attentional demand could be identified.

## Materials and Methods

### Participants

In this study, datasets of two previously reported experiments were merged and re-analyzed (Jaeger et al., 2020; Holtze et al., 2021), resulting in EEG recordings of forty-one German native participants (*N* = 20, mean age 22.45 years ± 2.74, 15 female; *N* = 21, mean age 24.19 years ± 3.93, 14 female). Participants were free of psychological or neurological conditions and had normal hearing abilities.

### Task

Both studies followed a similar procedure (for further details see Jaeger et al., 2020; Holtze et al., 2021). They contained multiple 10 min blocks of stimulus presentation. While the first block was the same for both studies, the studies differed in subsequent blocks. Therefore, only the first block was considered in the current study. The stimulus consisted of two simultaneously presented fairy tale audiobooks narrated in German. The tales were spoken by two different male speakers and were matched in sound intensity as described in Mirkovic et al. (2016). The same stimulus was used in both studies, but the studies differed in stimulus presentation. One used freefield audio presentation by positioning speakers to the front left and front right of the participants (±45°, Jaeger et al., 2020). The other used headphones, in particular behind-the-ear hearing aid dummies (Holtze et al., 2021). In this study, stimuli were preprocessed using a head-related impulse response to spatially separate both speakers (±30°, Kayser et al., 2009). Thus, one speaker was perceived from the front left while the other speaker was perceived from the front right. In both studies, participants were instructed by the experimenter to either attend to the left speaker (*N*_*Freefield*_ = 10; *N*_*Headphones*_ = 12) or the right speaker (*N*_*Freefield*_ = 10; *N*_*Headphones*_ = 9) while ignoring the other one throughout the whole experiment. Each participant was exposed to the same stimulus and experimental conditions only differed in the attentional allocation to either the story on the left or right side.

### EEG Recording

Participants were seated in a comfortable chair in a dimly lit and sound-attenuated booth. Two different cap configurations were used. In the freefield study a 96-channel Ag/AgCl cap (Easy-cap GmbH, Herschling, Germany) was used, while in the headphone study a 64-channel Ag/AgCl cap (Easy-cap GmbH, Herschling, Germany) was used. Cap electrodes around the ear were left unprepared due to the concurrent acquisition of ear-EEG signals using cEEGrids (Debener et al., 2015). This affected 12 electrodes in the freefield and ten in the headphone study. Furthermore, in the headphone study five electrodes in the neck region were left unprepared as they were not well attached to the scalp, leaving 84 and 49 prepared scalp electrodes in the freefield and headphone study, respectively. Both experiments recorded at a sampling rate of 500 Hz, used one EOG (electrooculography) electrode below each eye and a nose-tip reference electrode. Impedance was kept below 20 kΩ for all scalp electrodes. The EEG caps were connected to a stationary BrainAmp amplifier (Brainproducts GmbH, Gilching, Germany).

### EEG Preprocessing

The EEG data were analyzed using MATLAB (MATLAB R2020a, The Math-Works Inc. Natick, MA, United States) and the EEGLAB toolbox, version 2020.0 (Delorme and Makeig, 2004). First the data were screened for artifactual channels, which had a high standard deviation over time. For eight participants in the freefield study between 1 and 6 channels were rejected (mean = 3.4, *SD* = 2.1), mostly laying in the neck region. No channel in the headphone study was rejected. Afterward, the data were re-referenced to common average, low-pass filtered at 40 Hz (FIR filter, filter order: 100, window type: Hann), resampled to 250 Hz and high-pass filtered at 1 Hz (FIR filter, filter order: 500, window type: Hann). For further analysis, only the first 10 min block of stimulus presentation was considered, thus, for each participant a 10 min epoch after stimulus onset was created. For that, a constant delay between the EEG recording and the stimulus presentation was considered, based on the results obtained in a timing test.

The preprocessed data were cleaned from artifacts using the open source EEGLAB plugin *clean_rawdata* version 0.32 which automatically detects artifactual data using artifact subspace reconstruction (ASR). ASR is based on a sliding window principal that detects high-amplitude segments in relation to artifact-free calibration data. Each artifactual timepoint is removed and reconstructed based on the calibration data (Mullen et al., 2015). In the current study, no calibration data were recorded prior to the experiment. Therefore, to generate calibration data, the preprocessed data were cleaned using the ASR plugin functions *clean_drifts* [Transition: (0.25 0.75)] and *clean_windows* (MaxBadChan: 0.075). Except for the parameters in brackets, the default values were used. The generated calibration data were then submitted to the function *asr_calibrate* to compute the statistical properties of the calibration data and afterward submitted together with the preprocessed data to the function *asr_process*, which reconstructs the artifactual timepoints based on the calibration data.

Next, to match the channel layout of the freefield and headphone studies the interpolation function *interpmont* was used which is part of the open source Interpmont EEGLAB extension^1^. Lastly the preprocessed data were low-pass filtered at 15 Hz (FIR filter, filter order: 100, window type: Hann).

### Intersubject Correlation

ISCs were computed in MATLAB by adapting publicly available code^2^ that was written for the study conducted by Cohen and Parra (2016). ISC values were calculated using Correlated Component Analysis which finds components with maximal correlations between datasets (Dmochowski et al., 2012). For a detailed description of this method see Parra et al. (2019). In short, the within-subject cross-covariance (R_*w*_) and the between-subject cross-covariance values (R_*b*_) were computed. Then, the eigenvectors *v*_*i*_, where i = 1,…,D defines the dimensions, that capture the largest correlation of matrix R_*w*_^–1^R_*b*_ were calculated. R_*w*_ was regularized using a shrinkage parameter (γ = 0.4) before calculating the eigenvalues due to biased estimation of the eigenvalues for small R_*w*_ (Blankertz et al., 2011).

Synchrony in neural responses between participants within the same condition and between participants of opposite conditions were compared by calculating individual ISC values for each participant (Dmochowski et al., 2012; Cohen and Parra, 2016; Parra et al., 2019). Therefore, the EEG of each participant was once correlated with the EEG of all participants from the same attention condition (ISC_*same*_) and once with the EEG of all participants from the other attention condition (ISC_*other*_). For each participant *k*, ISC was calculated using the same projection vector *v*, which was computed from the data of all participants, excluding participant *k* to avoid over-fitting:

$$C_{k\,i}=\frac{v_{i}^{T}\,R_{b,k}\,v_{i}}{v_{i}^{T}\,R_{w,k}\,v_{i}}$$

where *R*_*b,k*_ represents the between-subject covariance and *R*_*w,k*_ the within-subject covariance:

$$R_{b,k}=\frac{1}{N-1}\,\sum_{k=1}^{N_{c}}\sum_{l=1,l\neq k}^{N_{c}}\left(R_{k\,l}+R_{l\,k}\right)$$

$$R_{w,k}=\frac{1}{N-1}\,\sum_{l=1,l\neq k}^{N_{c}}\left(R_{k\,k}+R_{l\,l}\right)$$

**FIGURE 1:**
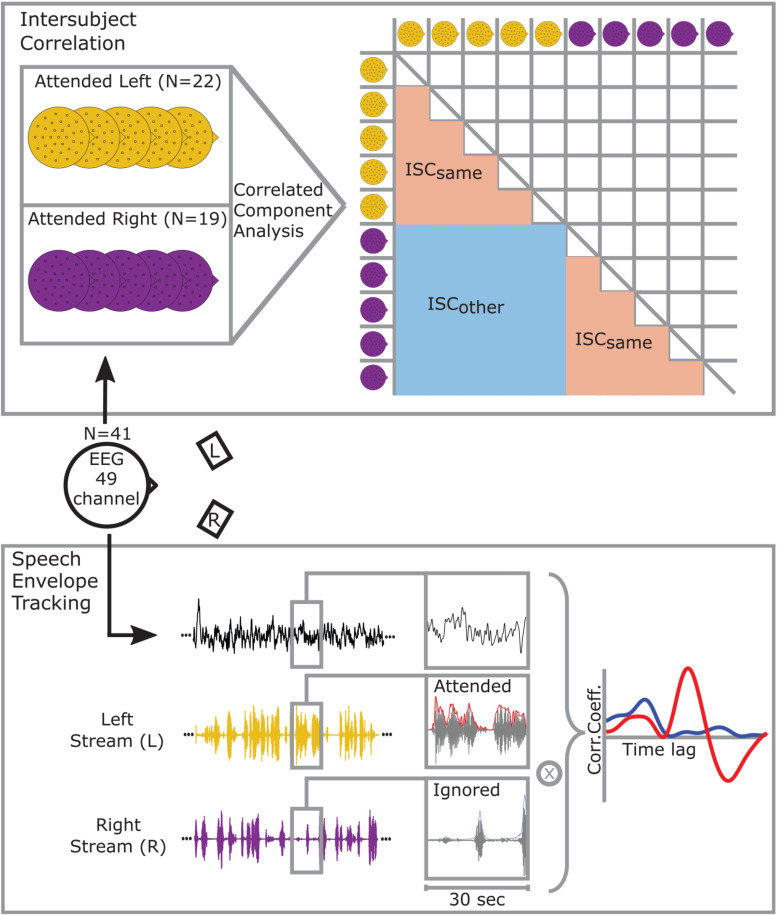
Participants were instructed to attend to one of two simultaneously presented stories while EEG was recorded. Experimental conditions differed in allocation of attention to the story presented from the front left (yellow) or front right (purple). A 49-channel configuration was used for further analysis. (Intersubject correlation) Correlations between participants’ EEG signals were maximized using Correlated Component Analysis. The matrix on the right illustrates that correlations between participants that also attended to the left or right story are categorized as ISC_*same*_ whereas correlations across conditions are categorized as ISC_*other*_. As the correlation matrix is symmetrical one half can be neglected. (Speech envelope tracking) Speech envelope tracking was performed by segmenting the signals into 30 s epochs. For each epoch, the EEG signal of every channel was cross-correlated with the attended (red) and ignored (blue) speech envelope. The figure shows an example when the left stream is attended to, and the right stream ignored.

R_*kl*_ is defined as the cross-covariances of all electrodes in participant *k* with all electrodes in participant *l* and is calculated as follows:

$$R_{k\,l}=\sum_{t}\left(x_{k}\,\left(t\right)-{\bar{x}}_{k}\right)\,{\left(x_{l}\,\left(t\right)-{\bar{x}}_{l}\right)}^{T}$$

where *x(t)* represents the scalp voltage at timepoint *t* = 1,…,S. *Nc* defines the number of participants to-be-correlated. When calculating ISC_*same*_, participant *l* attended to the same story as participant *k*, and *Nc* is the number of participants that also attended to the same story as participant *k*. Conversely, when calculating ISC_*other*_, participant *l* and participant *k* attended to different stories and *Nc* defines the number of participants that attended to the opposite story than participant *k*. In other words, ISC_*same*_ denotes the average correlation of participant *k* with people who attended to the same story and ISC_*other*_ denotes the average correlation of participant *k* with people who attended to the other story. If, for example, participant *k* attended to the left story, its averaged correlation value with people of the attend-left condition corresponds to ISC_*same*_ and attend-right condition to ISC_*other*_ (see Figure 1). Based on previous studies ISC values were summed over the three most correlating components to receive an ISC score (Dmochowski et al., 2012; Cohen and Parra, 2016; Ki et al., 2016; Cohen et al., 2018; Petroni et al., 2018).

To determine whether ISC values are above chance, a random ISC distribution was computed by randomly circulating the data of each participant in time. Thereby, the spatial and temporal structure of the EEG signal is preserved while any correlation between participants is random (Parra et al., 2019). The shifted data followed the same procedure as the original data, thus within- and between-subject covariance matrices were calculated to receive eigenvectors that capture the largest correlations. The eigenvectors were subsequently used to calculate the chance level of ISC_*same*_ and ISC_*other*_ as the sum over the three most correlating components. For each participant, calculating ISC on circular time shifted data was done over 100 iterations with different time shifts to generate a random distribution.

#### Classification

To replicate the findings of Ki et al. (2016) on the classification accuracy of ISC, we closely followed their analysis pipeline. The classification analyses aimed to discriminate between participants that attended to the left or the right story. Therefore, a projection vector was calculated for each condition (i.e., attended to left/right story) separately. Afterward, each participant was once correlated to all participants from the left side and once to all participants from the right side using the respective projection vector. The participant to-be-correlated was left out of the component extraction step, to avoid over-fitting. Individual correlations with the left group and the right group are further referred to as ISC_*left*_ and ISC_*right*_, respectively. Again, ISC scores were defined as the sum over ISC values with the three strongest correlated components. Next, the area under the receiver operator characteristic curve (AUC) was utilized to evaluate the prediction accuracy of ISC_*left*_ and ISC_*right*_ to correctly classify whether a participant attended to the left or the right side, respectively. Thus, two AUC models were created. One predicted whether or not a participant attended to the left story based on the ISC_*left*_ score and the other one predicted whether or not a participant attended to the right story based on the ISC_*right*_ score.

Chance-level AUC values were determined by randomly assigning the class labels (i.e., left/right) 1,000 times, resulting in a random AUC distribution. For each iteration, the procedure started with the extraction of the components for each group, continued with the calculation of individual ISC scores with the left and right group and ended with the calculation of AUC values.

#### Forward Model

Backward models, such as Correlated Component Analysis, find common sources of covariation in the observed data and store them into components (Parra et al., 2019). Thus, high-dimensional data like EEG recordings are reduced to a set of components. However, backward models cannot directly identify physiologically plausible brain activity patterns at the level of the scalp electrode (Parra et al., 2019). To fill this gap, the backward model was transformed into a forward model by recovering the observation from the component projection (Haufe et al., 2014).

### Speech Envelope Tracking

#### Speech Envelope

The speech envelope of each speech stream was extracted using a procedure described by Petersen et al. (2016). In short, the absolute of the Hilbert transform of the speech stream was taken and low-pass filtered at 15 Hz (FIR filter, filter order: 4, window type: Blackman). The first derivative of the filtered signal was taken to highlight tone and syllable onsets, and half-wave rectified. Finally, the signal was downsampled to 250 Hz to match the sampling frequency of the EEG signal.

#### Cross-Correlation

Speech envelope tracking was performed by cross-correlating the EEG signal with the speech envelopes. Therefore, each 10 min signal was epoched into twenty 30 s segments. Correlation coefficients were computed for each participant, channel, and epoch between the EEG signal and the speech envelope of the to-be-attended and to-be-ignored stream at time lags ranging from −500 to 500 ms (see Figure 1).

**FIGURE 2:**
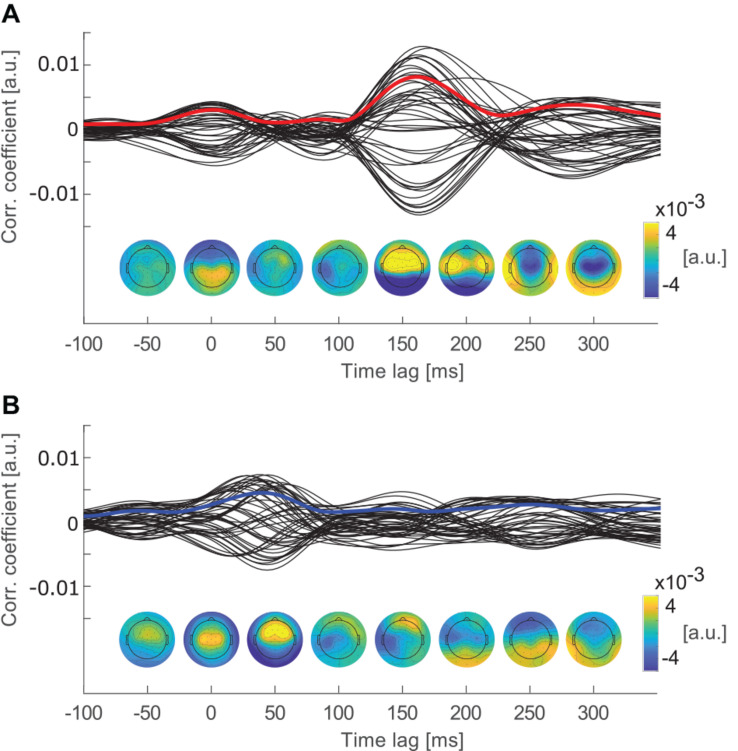
Cross-correlation functions of each channel averaged over epochs and participants. Topographies below show the channel weights, indicating channels that contributed most to the correlations at time lags from −50 to 300 ms in steps of 50 ms. **(A)** Cross-correlation functions of individual EEG channels with the attended speech envelope. The red line marks the global field power of the cross-correlation (GFP_*crosscorr*_). **(B)** Cross-correlation functions of individual EEG channels with the ignored speech envelope. The blue line marks GFP_*crosscorr*_ of the ignored speech envelope.

By averaging over epochs and participants, peak components with similar latencies and topographies as event related potential (ERP) components were revealed (Picton, 2013). A P2-like component was expected in the cross-correlation function of the attended stream between 100 and 200 ms time lag (e.g., Kong et al., 2014; Petersen et al., 2016). As the component was revealed by cross-correlation, it is further referred to as P2_*crosscorr*_ (Mirkovic et al., 2019; Jaeger et al., 2020). To account for individual differences in the spatial representations of the stimulus, the global field power (GFP) of the cross-correlation functions was estimated. Therefore, the standard deviation over all electrodes was calculated for the attended and ignored cross-correlation functions (see Figure 2). GFP describes the cross-correlation magnitude of all channels at different time lags and is, therefore, referred to as GFP_*crosscorr*_. The effect of attention on the GFP_*crosscorr*_ was investigated in the time window around P2_*crosscorr*_ which ranged from 130 to 184 ms time lag. This time window was determined by calculating the full width at half maximum of the P2_*crosscorr*_.

The individual selective attention effect on speech envelope tracking was calculated as follows. First, for each participant, the cross-correlation functions were averaged over all 20 epochs. Second, individual GFP_*crosscorr*_ functions for the attended and ignored stream were calculated. Third, the mean over the P2_*crosscorr*_ time window was taken, resulting in one value for the attended and ignored stream, respectively. Lastly, the value of the ignored stream was subtracted from the value of the attended stream.

### Statistical Analysis

All statistical analyses were done using RStudio (Version 1.2.5033; RStudio, Inc., Boston, MA, United States; R-Version: 3.6.1). For analyzing differences between ISCs, paired tests at an alpha level of 0.05 were used (Bonferroni corrected in case of multiple comparison). For normally distributed data, a paired *t*-test was used, and Cohens’ d was reported as an effect size. For non-normally distributed data, a Wilcoxon signed rank test was used and effect sizes are reported as correlation coefficients (*r*; Tomczak and Tomczak, 2014). Note that in the present work the same EEG data of an individual were used to calculate ISC values with the same and other attention condition. Thus, the initial observation (i.e., EEG recording) of the individual participant to-be-correlated was not independent for the two ISC scores. However, the participant pairs that constituted individual ISC_*same*_ and ISC_*other*_ values were unique, and efforts were made to increase the sample size by combining two datasets. We therefore argue that the influence of the individual observation only had a minor effect and that the use of dependent measures was reasonable.

Significance of the prediction accuracy was assessed by testing the original AUC value against the distribution of 1,000 class shuffled AUC estimates. For the random distribution, the AUC value at the significance level *p* < 0.001 (one-sided) was compared to the original accuracy, assuming that the real AUC value is higher than the 99.9th percentile of the permuted distribution (Combrisson and Jerbi, 2015). AUC values were calculated using the pROC package for R (Robin et al., 2011). To check for a difference in classification accuracies between the different models (i.e., ISC_*left*_ and ISC_*right*_) the two AUC values were tested against the null hypothesis that there is no difference between the classifications, using an equivalent to a Mann-Whitney *U*-test (DeLong et al., 1988).

Regarding speech envelope tracking it was first evaluated whether the selective attention effect on the cross-correlation was present. The selective attention effect was defined as the difference between the attended and ignored GFP_*crosscorr*_ functions in the P2_*crosscorr*_ time window. The difference was tested for significance using a paired Wilcoxon signed rank test (one-sided), because the values were not normally distributed. To investigate the relationship between ISC scores and the attention effect on cross-correlation, Spearmans rank correlation coefficient (rho) was calculated.

## Results

### Higher ISC Within a Condition Than Across Conditions

**FIGURE 3:**
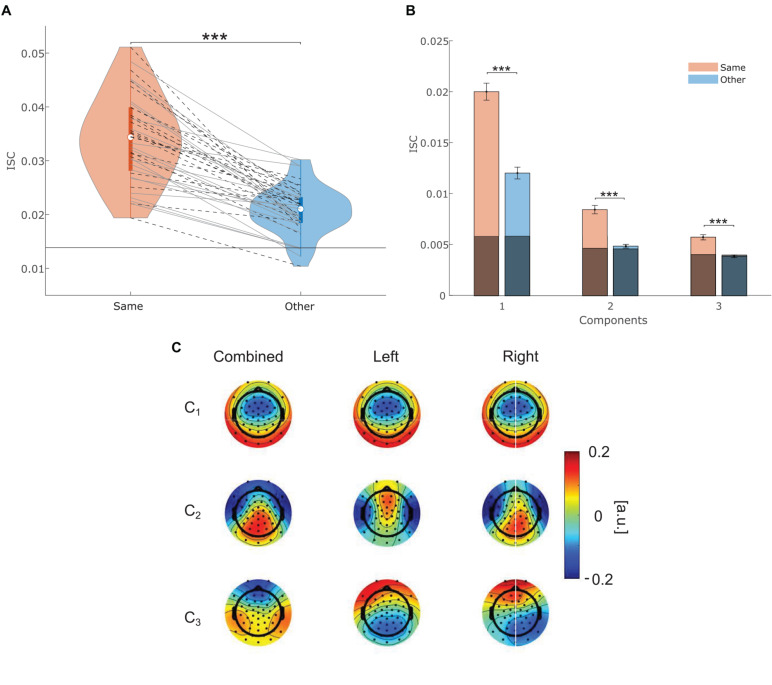
**(A)** Individual ISC scores between participants within the same attentional condition (red), and other attentional condition (blue). Participants from the freefield and headphone study are marked with solid gray and dashed black lines, respectively. The horizontal black lines represent 95th percentile of chance-level ISC scores for the same and other group correlation, respectively. **(B)** Mean and standard error of the mean (SEM) of intersubject correlations within a condition (same) and across conditions (other) for each component. 95th percentile of chance-level correlations is marked in gray. *** indicates significance at *p* < 0.001. **(C)** Forward model of the three most correlating components. The rows represent each component starting from the strongest in descending order. The first column contains data from all participants combined. The second and third column exclusively contain data from participants that attended to the left or right story, respectively. Channel weights which indicate channels that contribute most to the correlations between participants are indicated by color.

For each participant, two individual ISC scores were computed as the sum over the three components with the highest correlations. Individual ISC_*same*_ scores (*M* = 0.034, *SD* = 0.008) represent the magnitude in correlated EEG activity with participants that attended to the same story. ISC_*other*_ scores (*M* = 0.021, *SD* = 0.004) represents the magnitude in correlated EEG activity with participants that attended to the other story. A dependent t-test revealed that ISC_*same*_ scores were significantly higher than ISC_*other*_ scores [Figure 3A; *t*(40) = 11.845, *p* < 0.001], corresponding to a large effect size (Cohen’s *d* = 1.97). Apparently, all 41 participants showed a higher neural synchrony with people who attended to the same story than with people who attended to the other story. Furthermore, ISC scores were evenly distributed for the datasets of both studies, indicating that the differences in channel configuration and stimulus presentation between the two studies did not lead to a difference in ISC score distributions.

To test whether ISC scores are not merely chance-level correlations, ISC scores were compared against the chance-level distribution. As depicted in Figure 3A none of the ISC_*same*_ scores and only five individual ISC_*other*_ scores were within the 95th percentile range of the random distribution. Therefore, it can be concluded that all ISC_*same*_ and 87.8% of the ISC_*other*_ scores represent correlations above chance.

We also explored whether the effect of attention could be confirmed on individual ISC component values (see Figure 3B). Components are per definition independent from each other (Parra et al., 2019) and previous work has emphasized that the three components which contribute to ISC scores may reflect distinct neural processes (Dmochowski et al., 2012; Cohen and Parra, 2016; Ki et al., 2016). We found that correlations of the same and other condition were significantly different from each other on the first (V = 858, *p* < 0.001, *r* = 0.865), second [*t*(40) = 8.077, *p* < 0.001, Cohen’s *d* = 1.75], and third [*t*(40) = 6.79, *p* < 0.001, Cohen’s *d* = 1.34] component, even after correcting for multiple comparison (α = 0.05/3).

### Forward Model

The weights of the forward model represent the relationship between channels and component activity. They indicate channels that contributed much to the component, in other words to the correlation between participants. The spatial patterns shown in Figure 3C are similar to those reported in previous EEG-based ISC studies using continuous auditory stimuli (Cohen and Parra, 2016; Ki et al., 2016). Note that ISC_*same*_ and ISC_*other*_ values were based on the same projection vector. Therefore, they correspond to the spatial pattern calculated from all participants (first column), which appeared to switch order on the second and third component.

### Stronger Tracking of Attended Than Ignored Speech Envelope

**FIGURE 4:**
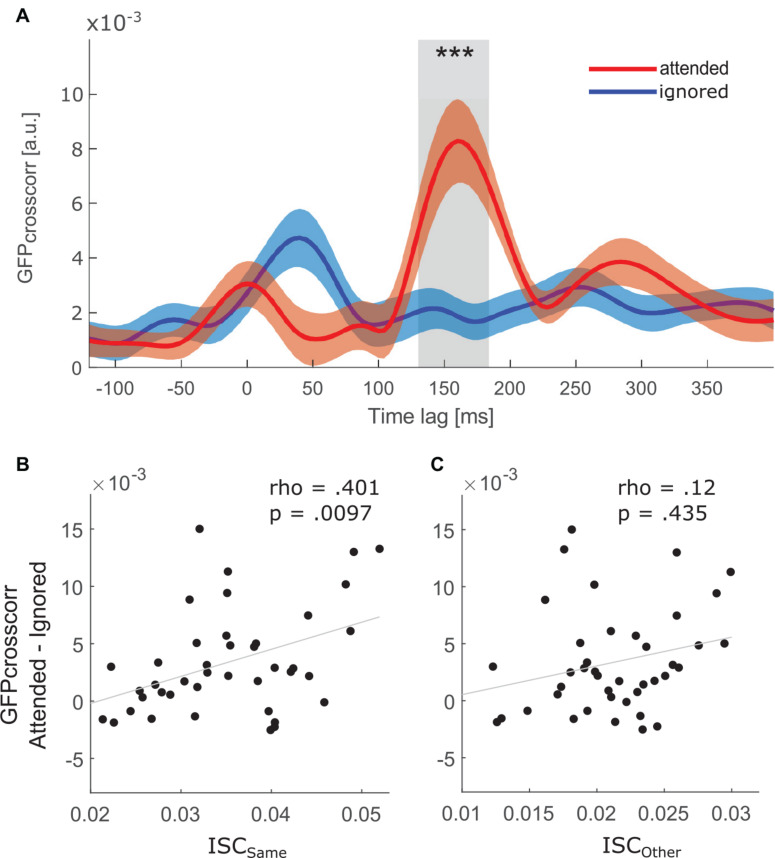
**(A)** Grand average global field power (GFP) of cross-correlation between the EEG signal and the attended (red) and ignored (blue) speech envelope after averaging over all epochs and participants. Red and blue shaded areas show the confidence interval of the respective function. *** indicates a difference (*p* < 0.001) between the two functions in the time window of P2_*crosscorr*_, i.e., from 130 to 184 ms time lag. (Bottom) Correlation between ISC and selective attention effect on speech envelope tracking. Attentional modulation of the speech envelope tracking is defined as the difference between the attended and ignored GFP_*crosscorr*_ in the P2_*crosscorr*_ time window. Each datapoint represents one participant. **(B)** Correlation with participant’s ISC_*same*_ score. **(C)** Correlation with participant’s ISC_*other*_ score.

Figure 4A depicts the global field power (GFP) of the cross-correlation between the EEG activity and the attended as well as ignored speech envelope. GFP_*crosscorr*_ of the attended stream was significantly larger than the GFP_*crosscorr*_ of the ignored stream in the P2_*crosscorr*_ time window (*V* = 746, *p* < 0.001, *r* = 0.639). In other words, a selective attention effect in the P2_*crosscorr*_ time window was present.

A Mann-Whitney *U*-test revealed no difference (*W* = 147, *p* = 0.95), in the selective attention effect on speech envelope tracking between the two studies that were included in the current study. Thus, there was no indication of sound presentation type or channel configuration influencing speech envelope tracking.

### ISC and Speech Envelope Tracking Are Related

The relationship between ISC and speech envelope tracking was assessed by correlating the difference between the attended and ignored GFP_*crosscorr*_ functions at the P2_*crosscorr*_ time window against ISC_*same*_ scores. As hypothesized, a significant positive correlation was found (Figure 4B, rho = 0.401, *p* = 0.0097). Thus, participants with greater attention modulation of speech envelope tracking showed a higher correlation with people who attended to the same stimulus. Furthermore, there was no relationship between ISC_*other*_ scores and the attention effect in the P2_*crosscorr*_ time window (Figure 4C, rho = 0.12, *p* = 0.435), suggesting that the neural synchrony between people attending to opposite stories is not related to the effect of attention observed in speech envelope tracking.

### High Prediction Accuracy for Attended Side

**FIGURE 5:**
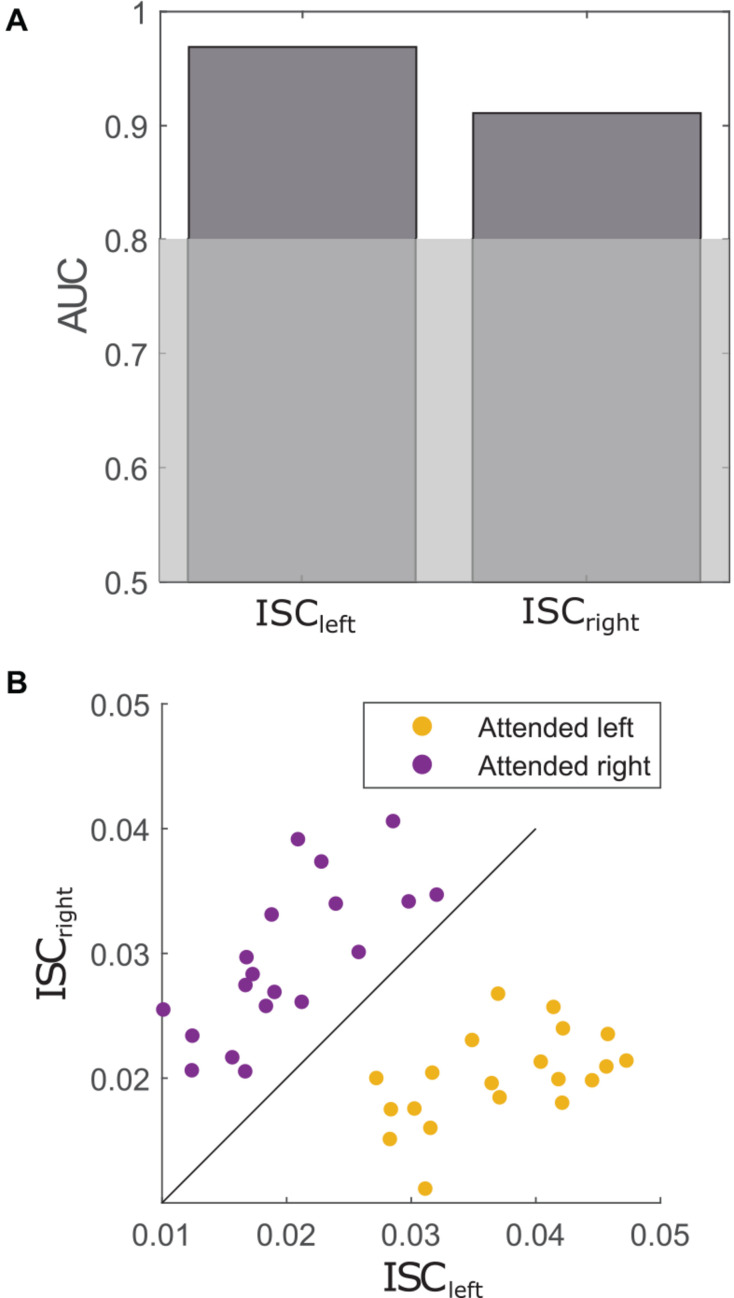
**(A)** Prediction accuracies assessed by AUC indicate the performance of ISC to correctly classify whether or not a participant attended to the left story (ISC_*left*_) or the right story (ISC_*right*_). Gray-shaded area indicates significance level at *p* < 0.001. **(B)** Each datapoint represents the relation of a participant’s ISC_*left*_ and ISC_*right*_ score. The colors indicate the condition of the participant. The diagonal line illustrates how informative each datapoint was for the models.

For classification, the condition of each participant (i.e., attended to the left/right story) was used as the ground truth. Each participant was correlated to other participants that attended to the left story (ISC_*left*_) or the right story (ISC_*right*_). These correlations were used to predict whether or not a given participant attended to the left or the right story, respectively. As depicted in Figure 5A ISC_*left*_ (AUC = 0.969) as well as ISC_*right*_ (AUC = 0.911) showed very high prediction accuracies above the significance level at *p* < 0.001. Thus, correlations with participants of one condition predicted with high accuracy whether or not a participant attended to a particular story. The AUC values showed no significant difference (*D* = 1.2, *p* = 0.234), suggesting that attention to either story did not alter the prediction accuracy. Figure 5B shows the relation between a participant’s ISC_*left*_ and ISC_*right*_ scores with respect to the condition. It appears that people who attended to the left story tend to show higher correlations to the left group than to the right group and vice versa.

## Discussion

We here evaluated whether selective auditory attention in the attended speaker paradigm affects the neural reliability between individuals, as measured by ISC. A strong difference in ISC scores was found when correlating participants that attended to the same vs. different story. We also confirmed the previously reported selective attention effect on speech envelope tracking. As hypothesized, this selective attention effect was positively correlated with ISC_*same*_ scores. Lastly, based on ISC scores, we were able to accurately predict to which story a participant attended to.

As expected, ISC scores were higher when a participant’s EEG was correlated with the EEG of those participants that attended to the same story (ISC_*same*_) compared to those that attended to the other story (ISC_*other*_). This attentional effect complements previous studies reporting higher ISC for attended compared to ignored stimuli (Ki et al., 2016; Cohen et al., 2018). However, the latter mentioned and other studies used a single uni- or multi-modal stream of information (Dmochowski et al., 2012; Cohen and Parra, 2016; Cohen et al., 2017; Iotzov et al., 2017). In contrast, in the current study, participants were exposed to two concurrently presented speech streams while they had to direct their attention to one of them, possibly suppressing the other. The participants’ EEG was correlated within the same condition as well as across conditions. Thus, attentional processing in the present work required disentangling two simultaneously perceived speech streams. The paradigm enabled us to look at the neural representations of both speech streams, which were represented in ISC_*same*_ and ISC_*other*_ scores, the difference of which reflecting selective auditory attention.

With regards to ISC between participants that attended to different stories (ISC_*other*_), most correlations were above chance. This implies that stimulus exposure alone, i.e., being presented with two stories simultaneously while focusing one’s attention on different stories, was sufficient to produce a shared response between participants. ISC_*same*_, on the other hand, represents synchronized brain activity in response to both stories with an additional attentional effect toward one story, which increased ISC (see Figure 3A). Importantly, the whole period of stimulus processing, i.e., 10 min, was reduced to two scores (ISC_*same*_ and ISC_*other*_). We interpret the difference between ISC_*same*_ and ISC_*other*_ scores as a participants’ attentional engagement with the to-be-attended story, over the entire 10 min. Engagement has previously been linked to ISC and has been described as a brain state with increased affect and attention (Dmochowski et al., 2012; Cohen et al., 2017, 2018). The current study follows a similar view of engagement as the mobilization of limited cognitive or neural resources that are necessary to comprehend a stimulus (Herrmann and Johnsrude, 2020). It follows that people who engage with the same stimulus, will have a more similar neural representation of the stimulus and, therefore, a high ISC score (Cohen et al., 2017; Nastase et al., 2019). A small difference between a participants’ ISC_*same*_ and ISC_*other*_ score suggests less engagement with the to-be-attended story compared to participants that had much higher ISC_*same*_ than ISC_*other*_ scores. However, all participants in the current study adhered to the instructions and engaged to some degree with the to-be-attended story, since higher ISC_*same*_ than ISC_*other*_ scores were found for all participants (see Figure 3A). Overall, the results are in congruence with previous studies on ISC and engagement (Dmochowski et al., 2012; Ki et al., 2016; Cohen et al., 2017) and further show that the neural reliability between participants hints at the direction of attentional engagement. However, as already mentioned, the strength of attentional modulation varied over participants. Behavioral measures could potentially disentangle whether this variability was due to individual differences in motivation to engage with the to-be-attended story or due to the capability to invest attentional resources (Herrmann and Johnsrude, 2020). As individuals differ regarding how well they can continuously attend to one story (Jaeger et al., 2020), measuring ISC over shorter, consecutive time periods during the experiment could also help to assess how fluctuations in engagement relate to the comprehension of the to-be-attended stream.

Looking at the single component level of ISC an attention effect could be confirmed on all three components (see Figure 3B). Interestingly, for ISC_*other*_ only the first component was clearly above chance. Given that ISC_*other*_ mostly captures the physical stimulus properties of both stories, we argue that the first component represents low-level sensory processing, which seems to contribute most to ISC scores. The forward models (see Figure 3C) for the first component were very similar regardless of whether all participants were considered together or separately for each condition (attention to the left or right story). They appeared to be similar to ISC forward models derived from multi-sensory conditions (Dmochowski et al., 2014; Cohen and Parra, 2016). We, therefore, conclude that this component primarily captures sensory processing. However, Cohen and Parra (2016) have emphasized that the first component may as well capture higher-level processes, as the common topography across stimulus modality may suggest involvement of brain areas beyond sensory processing. They further argue that the second component reflects auditory processing, due to a distinct topography compared to visual stimuli. On the contrary, Ki et al. (2016) have argued that the second and third component may also reflect higher-level processing. In the present study, ISC_*other*_ values were found to be close to chance for the second and third component while ISC_*same*_ values are clearly above chance. This indicates that these components captured neural activity that was not reliably produced across participants of different conditions. Furthermore, when considering participants that either attended to the left or the right story, the forward model shows similar topographies to previous work that included a single auditory stream (see Figure 3C; Cohen and Parra, 2016; Ki et al., 2016). However, compared to the participants that either attended to the left or right story the distribution of weights for the second and third component is different when all participants are considered together. Thus, the second and third component seem to represent processes that are specific to the attended stimulus. Future studies could investigate these components more specifically to draw stronger conclusion about the neural and cognitive processes that contribute to ISC.

Regarding speech envelope tracking, an attention effect, that is, a difference between the neural representation of the attended and ignored speech envelopes, was found between time lags ranging from 100 to 200 ms. This latency range is consistent with previous studies (Kong et al., 2014; Petersen et al., 2016). As predicted, a positive relationship was found between the strength of attentional modulation in speech envelope tracking and ISC scores between participants within the same condition. Note that the magnitude of ISC is related to the reliability of evoked neural activity within a participant (Ki et al., 2016; Parra et al., 2019). This is reflected in the current results as a reliable attention effect within a participant (speech envelope tracking) is related to the reliability of evoked response between a participant and others (ISC). Previous work has shown that the neural representation of stimulus features as captured with speech envelope tracking is related to ISC (Dmochowski et al., 2018; Kaneshiro et al., 2020). Our findings suggest that this relationship is partly driven by attention, since no relationship was found between attentional modulation of speech envelope tracking and ISC_*other*_ scores. In other words, the difference in the neural representation of the attended and ignored speech envelope was unrelated to the response across participants to the physical stimulus properties alone.

Speech envelope tracking and ISC are both influenced by the complexity of a stimulus. For example, audio-visual stimuli, compared to audio stimuli alone, result in higher ISCs (Cohen and Parra, 2016; Ki et al., 2016). Furthermore, the integration of audio-visual stimuli showed improved speech envelope tracking of the attended speaker compared to auditory stimuli alone (Zion Golumbic E. et al., 2013). Thus, multisensory stimulation seems to increase ISC as well as speech envelope tracking which fits to the positive relationship between the two methods observed in the current study. Overall, the findings could motivate future paradigms to take advantage of multisensory stimuli and move the field even further toward real-life stimulation.

Although the current study mainly focused on the investigation of ISC, one point should be mentioned regarding the global field power of the cross-correlation functions. An early difference (i.e., between 0 and 100 ms time lag) was observed between the cross-correlation functions of the attended and ignored speech envelope (see Figure 4A). At time lags close to and even before 0 ms, non-zero cross-correlation values were observed, which could be misinterpreted as a stimulus response before the stimulus actually started. However, this is likely a consequence of using a cross-correlation, which maps the speech envelope to the EEG signal at multiple overlapping timepoints, thereby causing temporal smearing (Crosse et al., 2016). Furthermore, at time lags earlier than 50 ms the ignored stream seemed to show a higher cross-correlation than the attended stream. It has previously been argued that this high cross-correlation of the to-be-ignored stream at early time lags is related to a subsequent suppression of that stream (Kong et al., 2014).

Correlating a participant’s EEG with the EEG of participants that attended to the left (ISC_*left*_) or right story (ISC_*right*_) strongly indicated to which story the participant attended to with prediction accuracies of 96 and 91%, respectively. These high prediction accuracies did not significantly differ from each other. The numerically higher prediction accuracy for ISC_*left*_ could be the result of subtle differences in sample size between the left and right condition. The calculated prediction accuracies exceeded those reported in a previous study that used a single speech stream (Ki et al., 2016). One of the reasons for this discrepancy could be related to the study design. To comprehend the to-be-attended story while a second interfering story is presented, one needs to utilize more cognitive resources than for listening to a single speech stream (Herrmann and Johnsrude, 2020). Thus, the attentional demand to engage with the to-be-attended story might have increased the synchrony between participants listening to the same story. In sum, we argue that the situational demand of the current study led to a high difference in ISC between people attending to and ignoring a story. Apparently, ISC not only marks engagement when the stimulus is attractive, but also when the situation is demanding.

The present study only considered task related top-down attentional processes and provided evidence that these processes have a strong influence on ISC. However, the natural environment is full of situations in which our attentional focus is captured by bottom-up, stimulus-driven events, such as salient sounds (Kayser et al., 2005) or a participant’s own name (Moray, 1959; Holtze et al., 2021). Certain scenes in a movie, such as a gun pointed at the viewer, elicit moments of high synchrony between participants (Dmochowski et al., 2012). Hence, salient events embedded in running speech streams might likewise synchronize participants’ neural responses. The degree to which these changes in attention also influence the reliability of evoked responses between participants remains yet unclear. Future studies considering the interplay of bottom-up and top-down attention could deepen our understanding of how humans process naturalistic stimuli.

As neuroscience moves toward ecologically valid paradigms, it becomes increasingly important to better understand the neural processing of complex stimuli. This study clearly demonstrated the potential of the ISC approach in capturing attentional engagement toward running speech in a scenario with multiple speakers. ISCs relate to individual neural representations of attended and ignored speech signals and help to dissociate between them. We conclude that shared responses between participants are informative about individual differences in attentional engagement and can help to understand the processing of complex stimuli in natural listening situations.

## Data Availability Statement

The data analyzed in this study is subject to the following licenses/restrictions: Requests to access these datasets should be directed to corresponding author MR, marc.rosenkranz@uni-oldenburg.de.

## Ethics Statement

The studies involving human participants were reviewed and approved by the Kommission für Forschungsfolgenabschätzung und Ethik, University of Oldenburg, Oldenburg, Germany. The patients/participants provided their written informed consent to participate in this study.

## Author Contributions

BH and MJ performed the data acquisition. MR analyzed the data and wrote the manuscript to which BH, MJ, and SD contributed with critical revisions. All authors approved the final version and agreed to be accountable for this work.

## Conflict of Interest

The authors declare that the research was conducted in the absence of any commercial or financial relationships that could be construed as a potential conflict of interest.

## Footnotes

1 https://github.com/jadesjardins/interpmont

2 https://www.parralab.org/isc/
